# Investigation of Anti-Inflammatory Potential of *N*-Arylcinnamamide Derivatives

**DOI:** 10.3390/molecules24244531

**Published:** 2019-12-11

**Authors:** Jan Hošek, Jiří Kos, Tomáš Strhársky, Lucie Černá, Pavel Štarha, Ján Vančo, Zdeněk Trávníček, Ferdinand Devínsky, Josef Jampílek

**Affiliations:** 1 Division of Biologically Active Complexes and Molecular Magnets, Regional Centre of Advanced Technologies and Materials, Faculty of Science, Palacký University, Šlechtitelů 27, 78371 Olomouc, Czech Republic; jan.hosek@upol.cz (J.H.); jiri.kos@upol.cz (J.K.); tomas.strharsky01@upol.cz (T.S.); lucie.cerna02@upol.cz (L.Č.); pavel.starha@upol.cz (P.Š.); jan.vanco@upol.cz (J.V.); zdenek.travnicek@upol.cz (Z.T.); 2 Faculty of Pharmacy, Comenius University, Odbojárov 10, 83232 Bratislava, Slovakia; 3 Department of Analytical Chemistry, Faculty of Natural Sciences, Comenius University, Ilkovičova 6, 84215 Bratislava, Slovakia

**Keywords:** cinnamamides, X-ray structure, polypharmacology, anti-inflammatory potential

## Abstract

A series of sixteen ring-substituted *N*-arylcinnamanilides, previously described as highly antimicrobially effective against a wide spectrum of bacteria and fungi, together with two new derivatives from this group were prepared and characterized. Moreover, the molecular structure of (2*E*)-*N*-(2-bromo-5-fluorophenyl)-3-phenylprop-2-enamide as a model compound was determined using single-crystal X-ray analysis. All the compounds were tested for their anti-inflammatory potential, and most tested compounds significantly attenuated the lipopolysaccharide-induced NF-κB activation and were more potent than the parental cinnamic acid. (2*E*)-*N*-[2-Chloro-5-(trifluoromethyl)phenyl]-3-phenylprop-2-enamide, (2*E*)-*N*-(2,6-dibromophenyl)- 3-phenylprop-2-enamide, and (2*E*)-*N*-(2,5-dichlorophenyl)-3-phenylprop-2-enamide demonstrated the highest inhibition effect on transcription factor NF-κB at the concentration of 2 µM and showed a similar effectiveness as the reference drug prednisone. Several compounds also decreased the level of TNF-α. Nevertheless, subsequent tests showed that the investigated compounds affect neither IκBα level nor MAPKs activity, which suggests that the *N*-arylcinnamanilides may have a different mode of action to prednisone. The modification of the C_(2,5)_ʹ or C_(2,6)_ʹ positions of the anilide core by rather lipophilic and bulky moieties seems to be preferable for the anti-inflammatory potential of these compounds.

## 1. Introduction

Inflammation is physiological immune reaction against infectious agents or injury, and its main role is to eradicate the noxious agent and to restore tissue homeostasis [1]. Under normal conditions, inflammation is self-limiting, but when it is uncontrolled and continuous, chronic inflammatory phase can develop subsequently. Chronic inflammation is a hallmark of many diseases, including atherosclerosis [2], rheumatoid arthritis [3], psoriasis [4], cancer [5], chronic respiratory diseases [6,7], and type 2 diabetes mellitus [8].

To modulate and drive the inflammatory response and inflammation itself are big tasks of current medicine. One way of handling such conditions is the application of natural compounds and their derivatives. Natural material is a huge source of bioactive compounds [9,10], and many currently used drugs are directly obtained from plants or fungi or are “nature-inspired” [11]. One such molecule is cinnamic acid (CA). It is a small organic molecule, which can be found in many kinds of plants in pure form or as a part of more complicated structures. CA and its natural and synthetic derivatives possess many interesting biological effects, such as antimicrobial [12,13], anticancer [14], anti-oxidant [13], and anti-inflammatory activities [15].

*N*-Arylcinnamides represent a group of synthetic derivatives of CA. This group of compounds possesses several potentially active moieties—styryl, amide (peptide-like [16]) bond, and aryl [17,18,19,20,21,22]. Thus, cinnamides can be considered as a privilege structure or a scaffold (a part) of more complex molecules in medicinal chemistry [23,24] The series of *N*-arylcinnamide derivatives presented in this work was previously tested for their anti-microbial activity (compounds **1**–**15** and **17**) [25,26], and three new derivatives **16** and **18** were prepared and characterized. Based on the concepts of polypharmacology, multifactorial diseases, and multitarget drugs [27], as well as the above-mentioned results, a group of eighteen *N*-arylcinnamanilides was chosen for the screening of their ability to moderate the inflammation-like reaction in vitro.

## 2. Results and Discussion

### 2.1. Chemistry

**Scheme 1:**
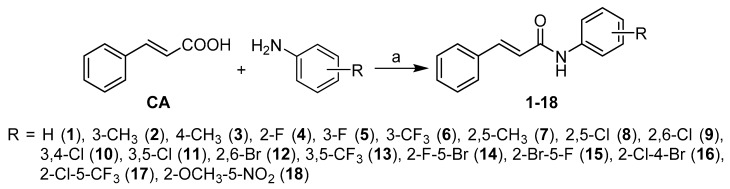
Synthesis of ring-substituted (2*E*)-*N*-aryl-3-phenylprop-2-enamides **1**–**18**. Reagents and conditions: (a) PCl_3_, chlorobenzene, MW, 20 min [25].

The investigated compounds (previously published compounds **1**–**15** and **17** [25] and two new compounds, **16** and **18**) were prepared using a one-step microwave-assisted synthesis, see Scheme 1. Briefly, the carboxyl group of CA was activated with phosphorus trichloride, and then the aminolysis of an acyl chloride by a ring-substituted aniline in dry chlorobenzene gave a final anilide. All the target derivatives were purified by recrystallization from ethanol. The yields of the target compounds ranged from 65% to 91%.

### 2.2. X-Ray Crystallography

**Figure 1:**
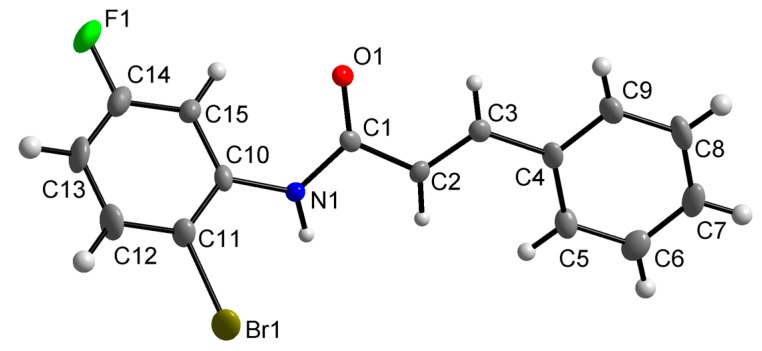
Molecular structure of (2*E*)-*N*-(2-bromo-5-fluorophenyl)-3-phenylprop-2-enamide (**15**). Non-H atoms are drawn as thermal ellipsoids at 50% probability level. H-atoms are not labelled and are displayed as balls in light-grey color.

**Figure 2:**
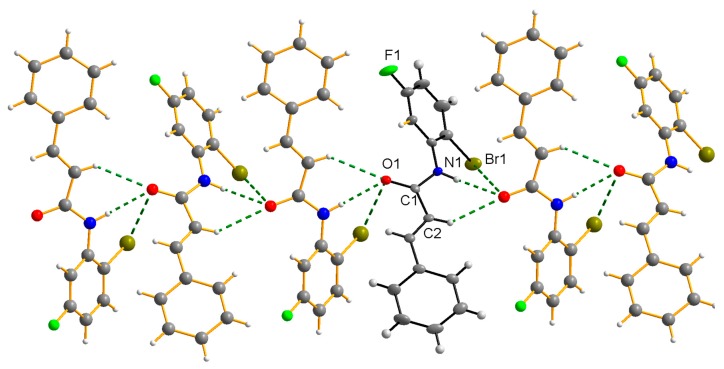
Part of the crystal structure of (2*E*)-*N*-(2-bromo-5-fluorophenyl)-3-phenylprop-2-enamide (**15**) showing N–H···O hydrogen bonds and Br···O non-covalent contacts (dashed green lines), connecting individual molecules into infinite polymeric chains.

Single crystal X-ray analysis revealed the molecular structure of (2*E*)-*N*-(2-bromo-5-fluorophenyl)-3-phenylprop-2-enamide (**15**), which is depicted in Figure 1. The crystal data and structure refinement for **15** are given in Table 1. The crystal structure consists of individual molecules, which are connected through strong N–H···O and weak C–H···O hydrogen bonds and via Br···O non-covalent contacts into 1D polymeric chains, see Figure 2 and Table 2. Moreover, the crystal structure is further stabilized by weak C–H···C hydrogen bonds and other non-covalent interactions of C···Br and C···C types connecting individual molecules into a 3D supramolecular structure. Both benzene rings (ring 1 = C4, C5, C6, C7, C8, C9, and ring 2 = C10, C11, C12, C13, C14, C15) are nearly ideally planar with the maximal deviation of 0.010(4) Å for the C11 atom from the least-squares planes fitted through the mentioned carbon atoms. The rings are mutually oriented by the dihedral angle of 47.0(2)°, and this value is substantially lower as compared to the values of 73.89(7)° and 79.46(7)° found for *N*-(2-fluorophenyl)cinnamamide [28] (in two crystallographically independent molecules with the asymmetric unit) and the value of 24.6(1)° found for *N*-(3-chlorophenyl)cinnamamide [29]. Moreover, there are also next two known structures of halogenated *N*-phenylcinnamamides, i.e., *N*-(3,5-difluoro-4-chlorophenyl)cinnamide and *N*-(3,4-difluorophenyl)cinnamide [30], in which both aromatic rings are almost coplanar, with the dihedral angle of 0.54° and 2.04°, respectively. The selected bond lengths and angles of compound **15** are given in Table S1 in Supplementary Materials. Interestingly, the C1–O1 bond length is significantly longer (1.312(6) Å) than that of a typical double C=O bond (an average value of 1.235(7) Å was found by the analysis of 39 structures containing the *N*-phenylcinnamamide skeleton deposited in CSD, ver. 5.40, Aug 2019 update). This lengthening of this bond in compound **15** may be connected with the involvement of the O1 atom into non-covalent bonding (see Figure 2).

### 2.3. In Vitro Cell Viability Assay

The panel of tested compounds was evaluated for their cytotoxicity against THP1-Blue™ NF-κB cells, see Table 3 and Figure S1 in Supplementary Materials for more information about dose-response curves. Compounds **10** (R = 3,4-Cl), **11** (R = 3,5-Cl), and **13** (3,5-CF_3_) showed the lowest IC_50_ values of 6.28 ± 2.32, 2.43 ± 1.06, and 2.17 ± 1.19 µM, respectively. The moderate cytotoxicity against THP1-Blue™ NF-κB cells was found in case of compound **6** (3-CF_3_) with IC_50_ = 11.60 ± 1.13 µM. All these compounds were also previously identified to have moderate influence on cell viability in different cancer cell lines [25].

The obtained results of cytotoxicity correlate with the log *k* and Clog *P* parameters calculated in the work of Pospisilova et al. [25]—the higher values of log *k* and Clog *P* parameters are, the higher IC_50_ values are, for example, compound **6** (log *k* = 0.4859, IC_50_ = 11.60 µM) >>>> compound **10** (log *k* = 0.6821, IC_50_ = 6.28 µM) >> compound **11** (log *k* = 0.8155, IC_50_ = 2.43 µM) ≈ compound **13** (log *k* = 0.9814, IC_50_ = 2.17 µM). Naturally, these observations correspond with distributive parameters π (see Table 3), a constant characterizing hydrophobicity (lipophilicity contribution) of individual moieties, substituents, and substructures in some skeleton [31,32]; compound **6** (π_Ar_ = 2.73) >>>> compound **10** (π_Ar_ = 2.77) >> compound **11** (π_Ar_ = 2.90) ≈ compound **13** (π_Ar_ = 3.98). Nevertheless, di-substitution with highly lipophilic and electron-withdrawing substituents (Cl or CF_3_) on the C_(3,5)_ʹ or C_(3,4)_ʹ positions of the anilide ring seems to be the most important for achieving a high antiproliferative effect. On the other hand, these facts correspond with the high antitubercular and antistaphylococcal potency of those agents as described recently [25].

### 2.4. Inhibition of NF-κB Activity and Cell Signaling In Vitro

**Figure 3:**
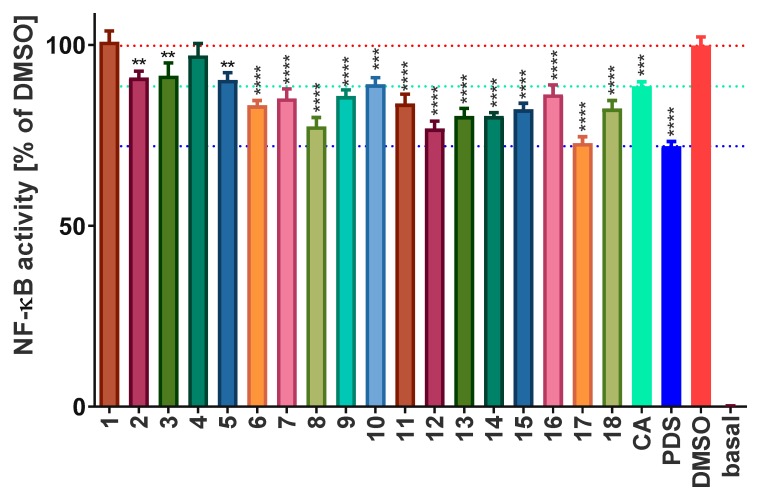
Effect of the tested compounds on the activity of transcription factor NF-κB. THP1-Blue™ NF-κB cells were pretreated by compounds (2 µM; or 0.5 µM for **10**, **11**, and **13**) or prednisone (PDS; 2 µM) dissolved in DMSO at indicated concentrations for 1 h. Then, lipopolysaccharide (LPS) 1 µg/mL was added to trigger the NF-κB activation. The activity of NF-κB was measured 24 h after LPS stimulation using QuantiBlue™ assay. Graph represents mean ± SEM. Red dotted line show value of DMSO-only treated group, azur dotted line CA treated group, and blue dotted line PDS treated group. ** indicates statistical significance (*p* < 0.01) to DMSO group; *** indicates statistical significance (*p* < 0.001) to DMSO group **** indicates statistical significance (*p* < 0.0001) to DMSO group.

Cinnamic acid alone and several CA derivatives demonstrated the ability to influence the activity or expression of the pro-inflammatory transcription factor NF-κB in previous studies [15,33]. The effect of *N*-arylcinnamamide derivatives with the modified cinnamyl phenyl ring on NF-κB activity was studied by Chen et al. [23] as well as by Jan et al. [34], but the effect of derivatives with the unchanged cinnamyl moiety is described here for the first time. Apart from compounds **1** and **4**, all anilides were able to significantly attenuate the lipopolysaccharide (LPS)-induced NF-κB activation by 10% to 27% in the non-toxic concentrations of 2 and 0.5 µM (used for compounds **10** (R = 3,4-Cl), **11** (R = 3,5-Cl), **13** (R = 3,5-CF_3_) with the lowest cell viability), see Figure 3. Moreover, most *N*-arylcinnamamide derivatives were more active than the parental CA. The highest inhibitory effect on NF-κB activity was observed for (2*E*)-*N*-[2-chloro-5-(trifluoromethyl)phenyl]-3-phenyl- prop-2-enamide (**17**), which was comparable with prednisone (PDS) applied at the same concentration level of 2 µM. The overall results indicate that monosubstituted *N*-arylcinnamanilides show lower inhibitory effect on NF-κB activity than the di-substituted ones. (2*E*)-3-Phenyl-*N*-[3-(trifluoromethyl)phenyl]prop-2-enamide (**6**), with bulky CF_3_ substitution of the anilide ring, is the only exception.

**Figure 4:**
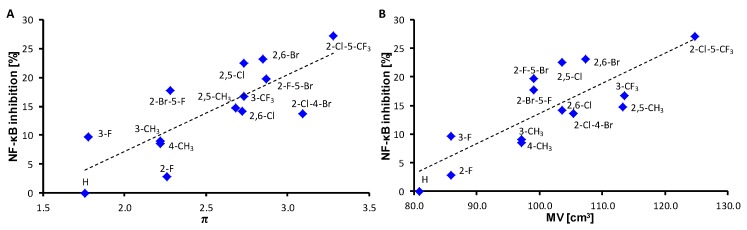
Dependence of NF-κB inhibition [%] on lipophilicity contributions of anilide rings expressed as distributive π parameters (**A**) and on molar volumes (MV [cm^3^]) of the whole anilide rings (**B**).

The electron σ parameters of individual substituents (listed in Pospisilova et al. [25]) play only a secondary role in this matter; however, it can be stated that the electron-withdrawing properties of the anilide substituent seem to be more advantageous. On the other hand, the position of the substituents on the anilide ring, lipophilicity, and bulkiness influence the activity of the compounds significantly. C_(2,5)_ʹ or C_(2,6)_ʹ di-substitutions by bulky substituents are preferred. The dependences of the inhibition of NF-κB on the lipophilicity expressed by distributive π parameters of the whole anilide ring and on the molar volume of the whole anilide ring are illustrated in Figure 4, where the discussed compounds are shown, with the exception of toxic compounds **10**, **11**, **13**, and compound **18** (R = 2-OCH_3_-5-NO_2_), which is excluded due to the completely different nature of the substituents compared to the rest (that are methyls or halogens). Figure 4A,B show the trends of the increasing ability to inhibit NF-κB with increasing distributive π parameters (correlation coefficient r = 0.7892, *n* = 14) as well as anilide ring bulkiness (r = 0.8118, *n* = 14).

**Figure 5:**
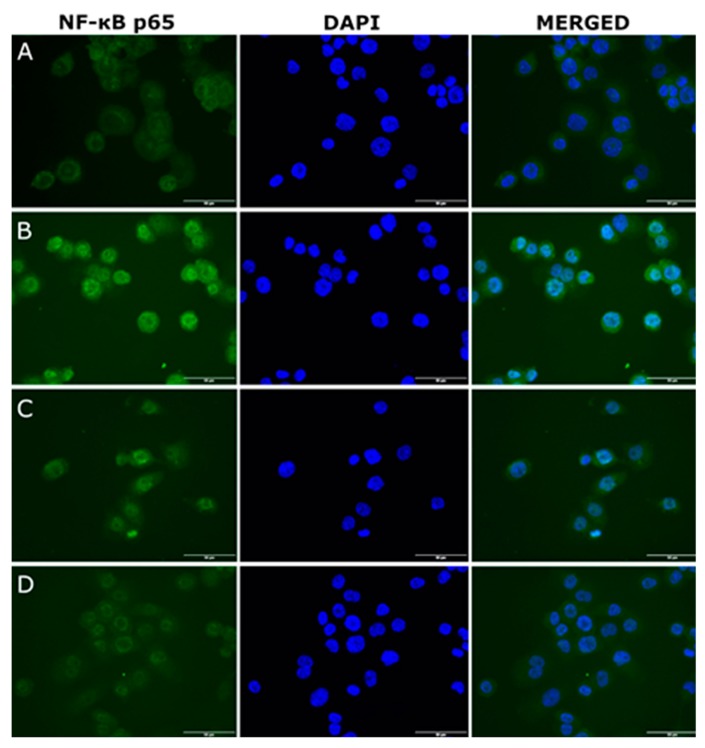
Effect of **17** (R = 2-Cl-5-CF_3_) and prednisone on the cellular distribution of transcription factor NF-κB. THP1-Blue™ NF-κB cells were pretreated by **17** 2 µM (**D**) or prednisone (PDS; 2 µM) (**C**), or by pure DMSO (**A**,**B**) for 1 h. Then, lipopolysaccharide (LPS) 1 µg/mL was added to activate the NF-κB (**B**–**D**). Control cell remained without LPS (**A**). The cellular distribution of NF-κB was observed 1 h after LPS stimulation using fluorescent microscope.

To confirm the effect of the most potent compound **17** on NF-κB, the analysis of its nuclear translocation was performed (see Figure 5). There is visible nuclear translocation of NF-κB after LPS stimulation. This movement was slightly affected by **17**. It is in agreement with observed inhibition of NF-κB activity and it can delineate the possible mechanisms of action.

**Figure 6:**
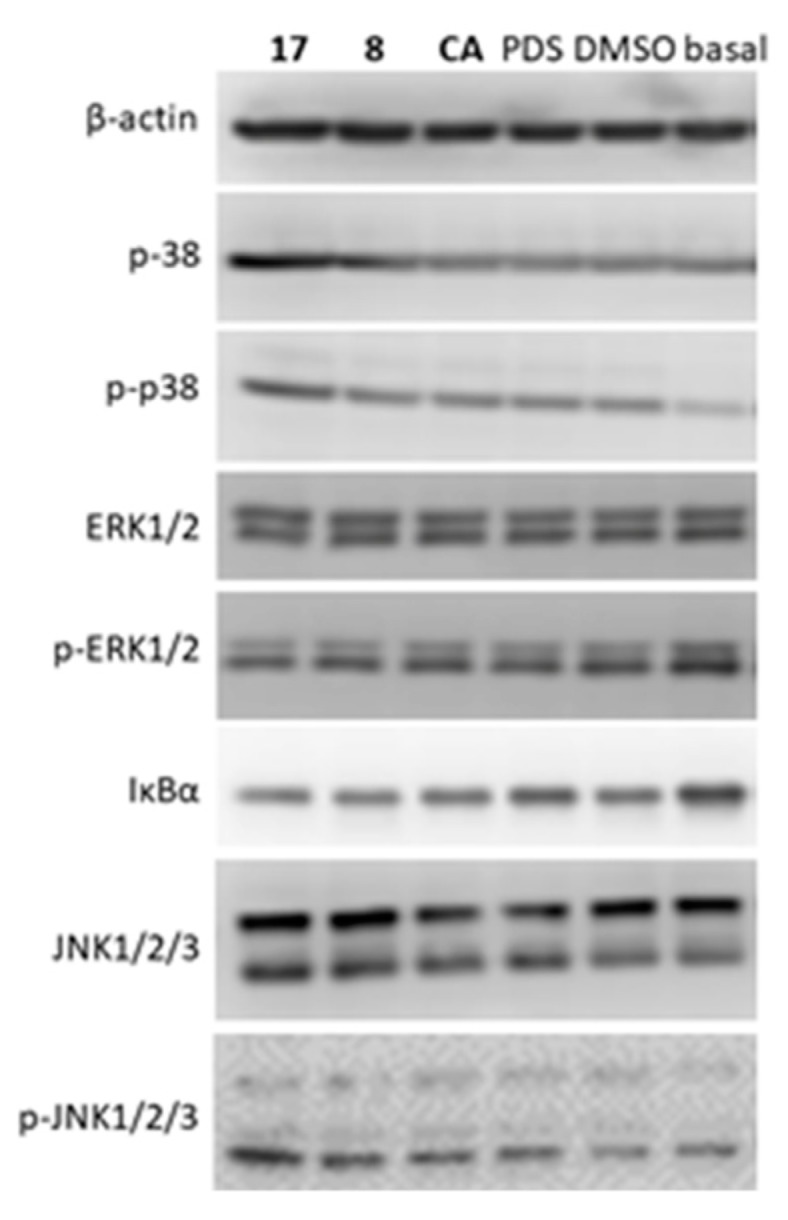
Effect of *N*-arylcinnamamide derivatives on the activation of MAPKs. THP1-Blue™ NF-κB cells were pretreated by compounds or PDS (2 µM) for 1 h, then LPS (1 µg/mL) was added to trigger the inflammatory signalization. Cells were collected 30 min after LPS stimulation and selected proteins were detected by Western blot and immunodetection.

Having discovered the high ability of compounds **17** (R = 2-Cl-5-CF_3_) and **8** (R = 2,5-Cl) to moderate the activity of NF-κB, we decided to elucidate further their mechanism of action and compare it with CA used as the reference compound. The activity of NF-κB is driven by the level of its inhibitor IκB and by the activity of several mitogen-activated protein kinases (MAPKs), especially p38 and c-Jun *N*-terminal kinase (JNK) [35,36], as well as ERK [37]. The influence of CA on NF-κB and MAPKs expression and activity was described previously [38,39], the same as for some cinnamides [23]. Our results showed no effect of compounds **17**, **8**, and CA on the IκBα levels and MAPKs activity, see Figure 6. Similar effects were also referred for a synthetic derivative of CA, WK2-16 ((*E*)-*N*-hydroxy-4-methoxy-2-(biphenyl-4-yl)cinnamide) [34,40]. It was proposed that WK2-16 could influence transcription factors YY-1 and STAT-1/-3. However, the discrepancy between the NF-κB inhibition and unobserved alteration of the above-mentioned signaling pathways could be also explained by a different mode of action as compared to known anti-inflammatory drugs, like PDS. It can be hypothesized that the tested *N*-arylcinnamanilides might act either via the inhibition of the nuclear translocation of NF-κB (as was described above), or influence its binding to DNA [41,42], or act by epigenetic regulation [43]. A combination of more than one mechanism of action is not excluded as well.

### 2.5. Inhibition of TNF-α Secretion

**Figure 7:**
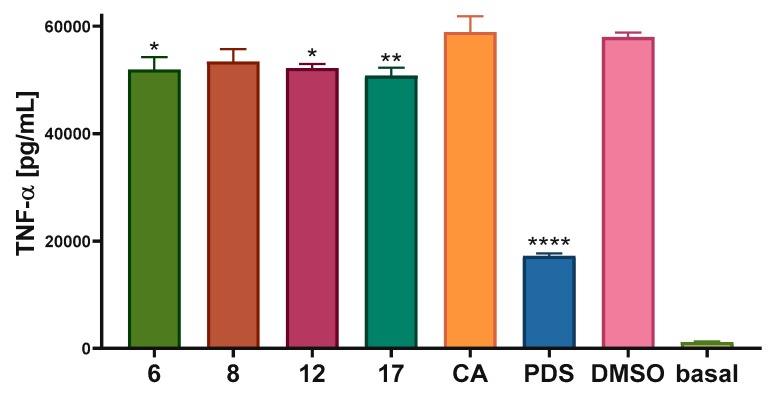
Effect of *N*-arylcinnamamide derivatives on the secretion of TNF-α. THP1-Blue™ NF-κB cells were differentiated to macrophages by PMA. Such cells were pretreated by compounds or PDS (2 µM) for 1 h, then LPS (1 µg/mL) was added to trigger the inflammatory signalization. Cultivation medium was collected 24 h after LPS stimulation, and the amount of TNF-α was measured by the ELISA technique. Graph represents mean ± SEM. * indicates statistical significance (*p* < 0.05) to DMSO group; ** indicates statistical significance (*p* < 0.01) to DMSO group **** indicates statistical significance (*p* < 0.0001) to DMSO group.

To verify the effect of tested compounds on NF-κB activity, the secretion of TNF-α, which is under its transcription control [44], was measured. CA is known for its ability to attenuate the production of this pro-inflammatory cytokine in vitro and in vivo [45,46]. In our experiment, pure CA did not affect the TNF-α production, see Figure 7. This could be caused by the usage of a very low, but pharmacologically relevant concentration of 2 µM. On the other hand, compounds **12** (R = 2,6-Br), **6** (R = 3-CF_3_), and **17** (R = 2-Cl-5-CF_3_) that showed the highest inhibitory effect on NF-κB were able to significantly decrease the level of TNF-α by 10%, 10.4%, and 12.4%, respectively. This correlates with the observed attenuation of NF-κB, when compound **17** reached the greatest effect. However, the effect of PDS was notably higher–the reduction of TNF-α by 70.3%. In case of NF-κB inhibition, the tested compounds are comparable with PDS, but PDS is more effective in TNF-α decreasing. This indicates that the modes of action of PDS and the discussed *N*-arylcinnamanilides are different.

## 3. Materials and Methods

### 3.1. Chemistry

#### 3.1.1. General Information

All reagents were purchased from Merck (Sigma-Aldrich, St. Louis, MO, USA) and Alfa (Alfa-Aesar, Ward Hill, MA, USA). Reactions were performed using a CEM Discover SP microwave reactor (CEM, Matthews, NC, USA). Melting points of **16** and **18** were determined on an apparatus STA 449 F1 Jupiter (NETZSCH, Selb, Germany). Infrared (IR) spectra were recorded on an UATR Zn/Se for a Spectrum Two™ Fourier-transform IR spectrometer (PerkinElmer, Waltham, MA, USA). The spectra were obtained by the accumulation of 32 scans with 4 cm^−1^ resolution in the region of 4000–400 cm^−1^. All ^1^H- and ^13^C-NMR spectra were recorded on a JEOL JNM-ECA 600II NMR spectrometer (600 MHz for ^1^H and 150 MHz for ^13^C, Jeol, Tokyo, Japan) in dimethyl sulfoxide-*d_6_* (DMSO-*d*_6_). ^1^H and ^13^C chemical shifts (δ) are reported in ppm. High-resolution mass spectra were measured using a high-performance liquid chromatograph Dionex UltiMate^®^ 3000 (Thermo Scientific, West Palm Beach, FL, USA) coupled with an LTQ Orbitrap XL^TM^ Hybrid Ion Trap-Orbitrap Fourier Transform Mass Spectrometer (Thermo Scientific) equipped with a HESI II (heated electrospray ionization) source in the positive mode.

#### 3.1.2. Synthesis

Cinnamic acid (3.37 mM) was suspended at room temperature in dry chlorobenzene (20 mL) inside a microwave tube, where phosphorus trichloride (1.7 mM) and the corresponding aniline derivative (3.37 mM) were added dropwise. Following this step, a magnetic stirrer was added to the tube and the reaction mixture was transferred to the microwave reactor at 120 °C for 20 min, where the synthesis at elevated pressure was performed. After the mixture was cooled to 60 °C, the solvent was evaporated in vacuum. A solid residue was washed with 2 M HCl, and a crude product was recrystallized, using 96% ethanol first, and then using 50% ethanol.

The *N*-arylcinnamanilides **1**–**15** and **17** were prepared and characterized previously by Pospisilova et al. [25].

*(2E)-N-(4-bromo-2-chlorophenyl)-3-phenylprop-2-enamide* (**16**). Yield 65%; Mp 169 °C; IR (cm^−1^): 3265, 3069, 3028, 1654, 1619, 1575, 1523, 1470, 1381, 1336, 1282, 1183, 969, 860, 825, 758, 709, 696, 647, 630, 567, 552, 506; ^1^H-NMR (DMSO-*d*_6_), δ: 9.57 (s, 1H), 7.93 (d, *J* = 8.7 Hz, 1H), 7.80 (d, *J* = 2.3 Hz, 1H), 7.66–7.60 (m, 3H), 7.57 (dd, *J* = 8.7 Hz, 2.3 Hz, 1H), 7.48–7.41 (m, 3H), 7,12 (d, *J* = 16 Hz, 1H), see Figure S2; ^13^C-NMR (DMSO-*d*_6_), δ: 164.01, 141.21, 134.63, 134.60, 131.64, 130.49, 130.01, 129.04, 127.89, 126.66, 121.57, 116.86, see Figure S3; HR-MS: [M + H]^+^ calculated 335.9785 *m*/*z*, found 335.9784 *m*/*z*, see Figure S4.

*(2E)-N-(2-methoxy-5-nitrophenyl)-3-phenylprop-2-enamide* (**18**). Yield 78%; Mp 209 °C; IR (cm^−1^): 3421, 3356, 1683, 1595, 1524, 1511, 1476, 1449, 1420, 1340, 1271, 1163, 1139, 1079, 1022, 978, 823, 769, 746, 710, 637, 591; ^1^H-NMR (DMSO-*d*_6_), δ: 9.74 (s, 1H), 9.22 (d, *J* = 2.7 Hz, 1H), 8.04 (dd, *J* = 9.1 Hz, 2.7 Hz, 1H), 7.66–7.60 (m, 3H), 7.48–7.40 (m, 3H), 7.30 (s, 1H), 7.27 (d, *J* = 6.9 Hz, 1H), 4.03 (s, 3H), see Figure S5; ^13^C-NMR (DMSO-*d*_6_), δ: 164.39, 154.10, 141.06, 140.37, 134.74, 129.95, 129.02, 128.03, 127.89, 122.08, 120.25, 115.38, 110.93, 56.79, see Figure S6; HR-MS: [M + H]^+^ calculated 299.1026 *m*/*z*, found 299.1020 *m*/*z*, see Figure S7.

### 3.2. X-Ray Crystallography

Crystals of (2*E*)-*N*-(2-bromo-5-fluorophenyl)-3-phenylprop-2-enamide (**15**) suitable for a single X-ray analysis were prepared by the slow evaporation of ethanol from the solution of compound **15** at 4 °C. The diffraction data were collected on a D8 QUEST diffractometer (Bruker) equipped with a PHOTON 100 CMOS detector, using Mo–Kα radiation (λ = 0.71073 Å). The data collection and reduction were performed using the APEX3 software [47]. The structure was solved by a direct method (SHELXS) and refined using the Bruker SHELXTL software package [48]. The benzene ring, involving C4, C5, C6, C7, C8, and C9 atoms, was refined with a riding group fitting (AFIX 66) with the fixed C–C bonds (1.39 Å). All H-atoms were found from difference Fourier maps and refined using a riding model, with C–H = 0.95 Å for (CH) and 0.88 Å for (NH) and with U_iso_(H) = 1.2 U_eq_(CH, NH). The graphics were drawn, and additional structural calculations were performed by the DIAMOND [49] software.

Crystallographic data has been deposited with the Cambridge Crystallographic Data Centre under CCDC deposition number 1957819. Copies of this information may be obtained free of charge from the Director, CCDC, 12 Union Road, Cambridge CB2 1EY, UK (fax: +44-1223-336033; e-mail: deposit@ccdc.cam.ac.uk or www: http://www.ccdc.cam.ac.uk).

### 3.3. Cell Cultivation

THP1-Blue™ NF-κB cell line was purchased from Invivogen (San Diego, CA, USA). Cells were cultured in RPMI 1640 medium (Merck, Darmstadt, Germany) containing stabilized 2 mM l-glutamine (Merck) supplemented with antibiotics [100 U/mL penicillin and 100 mg/mL streptomycin (Merck)] and 10% FBS (Merck). The cultures were passaged twice a week. Cells were kept in an incubator at 37 °C in a water-saturated atmosphere of air containing 5% CO_2_.

The viability of the used cell lines (5th–18th passage) was over 95% for each experiment. The cell number and viability were determined by staining with Trypan Blue solution. Cells were counted manually using a haemocytometer and an optical microscope. Cells that remained unstained were considered viable, while light red cells were considered non-viable. The viability percentage was calculated as the ratio of the number of all viable cells to the number of all cells.

### 3.4. Cell Viability Determination

The cell viability was determined as was described previously [50]. Briefly, THP1-Blue™ NF-κB cells (500,000 cells/mL) were washed by PBS, resuspended in a serum-free RPMI 1640 medium and seeded into 96-well plates (100 µL/well, i.e., 50,000 cells per each well). After 2 h, tested compounds (30–0.47 µM) dissolved in DMSO were added to the cells. The final concentration of DMSO was 0.1% (*v*/*v*) in each well. Measurements were taken after 24 h incubation with the tested substances. Viability was measured by the Cell Proliferation Reagent kit WST-1 (Roche Diagnostics, Basel, Switzerland) according to the manufacturer’s manual. The amount of formazan formed, which corresponded to the number of metabolically active cells in the culture, was calculated as a percentage of the control cells, which were treated only with serum-free RPMI 1640 medium and were assigned as 100%. The IC_50_ values were calculated by four-parameter logistic (4PL) analysis from obtained viability curves.

### 3.5. Determination of NF-κB Activity

The activity of NF-κB transcription factors was evaluated on THP1-Blue™ NF-κB cells as described previously [50,51]. Briefly, cells were transferred into serum-free RPMI 1640 medium as was delineated above in Section 3.4. All tested compounds were added 2 h later in non-toxic concentration 2 µM but compounds **10**, **11**, and **13** in concentration 0.5 µM. After 1 h of incubation of the treated cells with the samples, cells were stimulated by lipopolysaccharide (LPS) from *E. coli* 0111:B4 (Merck) dissolved in serum-free RPMI 1640 medium (1 µg/mL). After 24 h of incubation, 20 µL of cultivation medium was mixed with 200 µL of Quanti-Blue™ medium (Invivogen) and incubated according to the manufacturer’s instructions at 37 °C for 30–40 min. The activity of NF-κB was determined as secretion of embryonic alkaline phosphatase spectrophotometrically in a Tecan Infinite M200 (Tecan, Männedorf Switzerland) at 650 nm and compared with an untreated control (100%).

### 3.6. Immunocytochemical Analysis of NF-κB Nuclear Migration

To differentiate THP1-Blue™ NF-κB monocytes into macrophages, the cells were stimulated with phorbol myristate acetate (PMA) at the final concentration of 50 ng/mL, as was described previously [51,52]. Cells were seeded into 8-well cell culture glass slide (SPL, Korea) at the concentration of 500,000 cells/mL, 150,000 cells per well. The compound **17** was added at the final concentration of 2 µM, and 1 h later, the cells were activated by LPS (1 µg/mL). After 1h, the culture slide was washed with PBS and fixed with 4% formaldehyde. The cells were permeabilized by 0.1% Triton X-100 (*v*/*v*; Merck) and blocked with 3% bovine serum albumin (BSA, Merck) dissolved in PBS. The primary antibody NF-κB p65 (Abcam, Cambridge, UK; product No. ab16502) at concentration 2.5 µg/mL was used for overnight incubation at 4 °C. After that, secondary anti-rabbit antibody labeled with Alexa Fluor^®^ 488 (Cell Signaling; product No. 4412; dilution 1:1000) was added for 1 h in dark, and the nuclei were stained using 0,5 µg/mL DAPI (Merck) for 5 min. The wells were washed three-times between the mentioned steps with PBS. The coverslip was mounted with VectaShield mounting (Vector laboratories, Burlingame, CA, USA) medium and sealed with the nail polish. The nail polish was allowed to dry at 4 °C and the translocation of NF-κB was observed by fluorescence microscopy.

### 3.7. Signaling Pathway Analysis

The level of IĸB and the amount of activated (phosphorylated) mitogen-activated protein (MAP) kinase p38, ERK1/2, and JNK was determined on the THP1-Blue™ NF-κB, in a way similar to that described previously [53]. Briefly, cells cultivated in serum-free medium were pretreated with the tested compounds (**8**, **17**, cinnamic acid, or prednisone) at concentration 2 µM. One hour later, 1 µg/mL of LPS was added, and after 30 min the cells were collected into ice-cold Mammalian Cell Lysis Buffer (Abcam, Cambridge, UK) with phosphatase inhibitors PhosSTOP (Roche, Mannheim, Germany). Cell lysates were centrifuged 3000 g/5 min/4 °C and supernatants were mixed with 5× Laemmli reducing buffer [250 mM Tris-HCl pH 6.8, 10% (*w*/*v*) SDS, 30% (*v*/*v*) glycerol, 5% (*v*/*v*) β-mercaptolethanol, 0.04% (*w*/*v*) bromphenol blue], and incubated at 95 °C for 5 min. To separate the proteins, 10 μg of the proteins was loaded onto a 12% polyacrylamide gel and then transferred electrophoretically to PVDF (polyvinylidene fluoride) membranes that were subsequently blocked using 5% BSA dissolved in TBST buffer [150 mM NaCl, 10 mM Tris base, and 0.1% (*v*/*v*) Tween-20]. The membranes were incubated at 4 °C for 16 h with primary anti-IĸB-α (Cell Signaling; product No. 4814; dilution 1:1,000), anti-p44/42 MAPK (ERK1/2) (Cell Signaling; product No. 9102; dilution 1:1,000), anti-phospho-p44/42 MAPK (ERK1/2) (Cell Signaling; product No. 9101; dilution 1:1,000), anti-JNK1/2/3 (Abcam; product No. ab179461; dilution 1:1,000), anti-phosho-JNK1/2/3 (Abcam; product No. ab76572; dilution 1:5,000), anti-p38 MAPK (Cell Signaling; product No. 8690; dilution 1:1,000), anti-phospho-p38 MAPK (Cell Signaling; product No. 9210; dilution 1:1,000), and anti-β-actin (Santa Cruz, Aachen, Germany; product No. sc-47778; dilution 1:5000) antibodies. After washing, the secondary anti-mouse and anti-rabbit IgG antibodies (Cell Signaling, products No. 7076S and 7074P2) were diluted 1:2000 and applied to the membranes, which were incubated for 1 h at the laboratory temperature. The amount of bound secondary antibody was determined using WesternSure^®^ PREMIUM Chemiluminescent Substrate (LI-COR, Lincoln, NE, USA). Chemiluminescence was detected using a LI-COR C-DiGit chemiluminescence imaging system (LI-COR).

### 3.8. Differentiation Monocytes into Macrophages and Evaluation of TNF-α Secretion

Differentiated THP1-Blue™ NF-κB macrophages (see the chapter 3.6) were pre-treated for 1 h with 2 µM solutions of the tested compounds **8**, **12**, **6**, **17**, cinnamic acid, and prednisone dissolved in DMSO, or only the vehicle [0.1% (*v*/*v*) DMF solution]. The inflammatory-like response was triggered by adding 1 μg/mL of LPS from *E. coli* 0111:B4 (Sigma-Aldrich) to the pre-treated macrophages; the control cells were left without LPS treatment. After 24 h, the supernatants were collected and the concentration of TNF-α was measured using a Human TNF-α ELISA Kit (Diaclone, Besançon, France), according to the manufacturer’s manual.

### 3.9. Statistical Analysis

Statistical analysis was carried out in GraphPad Prism 8.0.1 (San Diego, CA, USA). Outliers were identified by the ROUT statistical method (Q = 5%) and excluded for further analysis. Data were expressed as mean ± SEM. Groups were compared with the help of the one-way ANOVA test followed by Fisher’s LSD multiple comparison test. The value *p* < 0.05 was assigned as statistically significant.

## 4. Conclusions

A series of eighteen ring-substituted *N*-arylcinnamanilides was designed. The molecular structure of (2*E*)-*N*-(2-bromo-5-fluorophenyl)-3-phenylprop-2-enamide (**15**) as a model compound was determined by single-crystal X-ray diffraction. All the compounds were screened for their anti-inflammatory potential at the pharmacologically relevant, non-toxic concentration of 2 µM, only least viable compounds **10** (R = 3,4-Cl), **11** (R = 3,5-Cl), and **13** (3,5-CF_3_), which IC_50_ ranged from 2.17 to 6.28 µM on THP1-Blue™ NF-κB cell line, were tested at the concentration level of 0.5 µM. It seems that cell viability is mainly affected by di-substitution on the C_(3,5)_ʹ or C_(3,4)_ʹ positions by Cl and/or CF_3_, i.e., groups with highly lipophilic and strong electron-withdrawing properties. Thus, although it would be advantageous to obtain compounds with both antimicrobial and anti-inflammatory activity, compounds **10**, **11**, and **13** were excluded from further study because of this undesirable cytotoxic effect. Most tested compounds significantly attenuated the LPS-induced NF-κB activation. They were more potent than the parental cinnamic acid. (2*E*)-*N*-[2-Chloro-5-(trifluoromethyl)phenyl]- 3-phenylprop-2-enamide (**17**), (2*E*)-*N*-(2,6-dibromophenyl)-3-phenylprop-2-enamide (**12**), and (2*E*)-*N*-(2,5-dichlorophenyl)-3-phenyl-prop-2-enamide (**8**) demonstrated the highest activity within the series that was comparable with prednisone. Compounds **12** and **17** also decreased the level of TNF-α, which correlates with the observed attenuation of NF-κB. On the other hand, subsequent tests showed that the investigated compounds did not affect IκBα level and MAPKs activity as expected. Based on above-mentioned fact, it can be hypothesized that the investigated *N*-arylcinnamanilides may have a different mode of action, which may consist of the nuclear translocation of NF-κB inhibition, or affect its binding to DNA, or act by epigenetic regulation, while the combinations of these effects are not excluded. In conclusion, it can be stated that anti-inflammatory activity (while minimizing antiproliferative activity) is positively influenced by di-substitution on the C_(2,5)_ʹ or C_(2,6)_ʹ positions by rather lipophilic and preferably bulky moieties that causes the anilide ring to rotate, resulting in a non-planar configuration of the entire system, as confirmed by the crystal structure.

## Figures and Tables

**Table 1:** Crystal data and structure refinement for compound **15**.

Formula	C_15_H_11_BrFNO
Formula weight	320.16
Temperature	150(2) K
Wavelength	0.71073 Å
Crystal system	Monoclinic
Space group	*P*2_1_/c
Unit cell dimensions	*a* = 9.410(3) Å, *α* = 90°
	*b* = 14.645(5) Å, *β* = 108.633(15)°
	*c* = 10.184(4) Å, *γ* = 90°
Volume	1329.9(8) Å^3^
*Z*	4
Density (calculated)	1.599 g/cm^3^
Absorption coefficient	3.093 mm^−1^
*F*(000)	640
Crystal size	0.140 × 0.140 × 0.060 mm
Theta range for data collection	2.284 to 24.999°
Index ranges	−11 ≤ *h* ≤ 11, −17 ≤ *k* ≤ 17, −12 ≤ *l* ≤ 12
Reflections collected	22311
Independent reflections	2339 [R(int) = 0.1233]
Absorption correction	Semi-empirical from equivalents
Refinement method	Full-matrix least-squares on *F*^2^
Data/restraints/parameters	2339/0/160
Goodness-of-fit on *F*^2^	1.067
Final *R* indices [I > 2σ(I)]	*R*_1_ = 0.0523, *wR_2_* = 0.1295
*R* indices (all data)	*R*_1_ = 0.0746, *wR*_2_ = 0.1391
Largest diff. peak and hole	1.162 and −1.065 e.Å^−3^

**Table 2:** Parameters of selected non-covalent contacts (in Å and °) in the crystal structure of compound **15**.

D-H···A	d(D-H)	d(H···A)	d(D···A)	<(DHA)
N(1)-H(1)···O(1)(i)	0.88	2.20	3.065(5)	167.5
C(2)-H(2) ···O(1)(i)	0.95	2.71	3.507(6)	141.8
Br(1)···O(1)(i)	–	–	3.346(3)	–

Symmetry transformations used to generate equivalent atoms: (i) x, −y+1/2, z+1/2.

**Table 3:** List of ring-substituted *N*-arylcinnamanilides **1**–**18** and their influence on the viability of THP1-Blue™ NF-κB cell line (IC_50_ [µM] ± SEM of three independent measurements), NF-κB inhibition [%] in comparison with the starting cinnamic acid (CA), predicted values of distributive parameters π, and molar volume (MV [cm^3^]) of anilide (Ar).

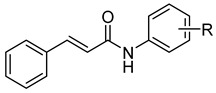					
Comp.	R	IC_50_ [µM] ± SEM	NF-κB inhibition [%]	π_Ar_^a^	MV ^a^ [cm^3^]
**1**	H	>30	0 ^b^	1.76	80.88
**2**	3-CH_3_	>30	9.00 ^b^	2.22	97.11
**3**	4-CH_3_	>30	8.50 ^b^	2.22	97.11
**4**	2-F	>30	2.87 ^b^	2.26	85.90
**5**	3-F	>30	9.67 ^b^	1.78	85.90
**6**	3-CF_3_	11.60 ± 1.13	16.67 ^b^	2.73	113.57
**7**	2,5-CH_3_	>30	14.78 ^b^	2.68	113.27
**8**	2,5-Cl	>30	**22.50** ^b^	2.73	103.54
**9**	2,6-Cl	>30	14.11 ^b^	2.72	103.54
**10**	3,4-Cl	6.28 ± 2.32	10.89 ^c^	2.77	103.54
**11**	3,5-Cl	2.43 ± 1.06	16.22 ^c^	2.90	103.54
**12**	2,6-Br	>30	**23.12** ^b^	2.85	107.33
**13**	3,5-CF_3_	2.17 ± 1.19	19.67 ^c^	3.98	145.57
**14**	2-F-5-Br	>30	19.67 ^b^	2.87	99.09
**15**	2-Br-5-F	>30	17.78 ^b^	2.28	99.09
**16**	2-Cl-4-Br	>30	13.67 ^b^	3.09	105.44
**17**	2-Cl-5-CF_3_	>30	**27.12** ^b^	3.28	124.72
**18**	2-OCH_3_-5-NO_2_	>30	17.56 ^b^	1.12	115.77
**CA**	–	>30	11.44 ^b^	–	–

^a^ calculated using ACD/Percepta ver. 2012 (Advanced Chemistry Development, Inc., Toronto, ON, Canada), ^b^ concentration of compounds 2 µM, ^c^ concentration of compounds 0.5 µM.

## Supplementary Materials

The supplementary materials are available online.
