# Supplementary material for: Investigation of Anti-Inflammatory Potential of N-Arylcinnamamide Derivatives
Source: Molecules. 2019 Dec 11;24(24):4531. doi: 10.3390/molecules24244531 (PMC6943612; doi:10.3390/molecules24244531)
Supplement: Supplementary file 1 [file molecules-24-04531-s001.pdf]

Supplementary Materials

Investigation of Anti-inflammatory Potential of *N*-Arylcinnamamide Derivatives

Jan Hošek [1], Jiří Kos [1], Tomáš Strhársky [1], Lucie Černá [1], Pavel Štarha [1], Ján Vančo [1], Zdeněk Trávníček [1], Ferdinand Devínsky [2,*], and Josef Jampílek [1,3,*]

[1] Division of Biologically Active Complexes and Molecular Magnets, Regional Centre of Advanced Technologies and Materials, Faculty of Science, Palacký University, Šlechtitelů 27, 78371 Olomouc, Czech Republic; jan.hosek@upol.cz (J.H.); jiri.kos@upol.cz (J.K.); tomas.strharsky01@upol.cz (T.S.); lucie.cerna02@upol.cz (L.Č.); pavel.starha@upol.cz (P.Š.); jan.vanco@upol.cz (J.V.); zdenek.travnicek@upol.cz (Z.T.)

[2] Faculty of Pharmacy, Comenius University, Odbojárov 10, 83232 Bratislava, Slovakia

[3] Department of Analytical Chemistry, Faculty of Natural Sciences, Comenius University, Ilkovičova 6, 84215 Bratislava, Slovakia

* Correspondence: fdevinsky@gmail.com (F.D.); josef.jampilek@gmail.com (J.J.)

Table S1. Selected bond lengths (Å) and angles (°) in (2*E*)-*N*-(2-bromo-5-fluorophenyl)-3-phenylprop-2-enamide (**15**).

Br(1)-C(11)	1.896(5)
F(1)-C(14)	1.353(6)
O(1)-C(1)	1.312(6)
N(1)-C(1)	1.399(6)
N(1)-C(10)	1.400(6)
C(1)-C(2)	1.488(6)
C(2)-C(3)	1.352(7)
C(3)-C(4)	1.487(5)
C(4)-C(5)	1.3900
C(1)-N(1)-C(10)	122.7(4)
O(1)-C(1)-N(1)	126.0(4)
O(1)-C(1)-C(2)	124.8(4)
N(1)-C(1)-C(2)	109.2(4)
C(3)-C(2)-C(1)	117.3(4)
C(2)-C(3)-C(4)	123.0(4)
C(5)-C(4)-C(3)	123.8(3)
C(10)-C(11)-Br(1)	119.8(4)
C(12)-C(11)-Br(1)	119.9(4)
C(13)-C(14)-F(1)	117.5(5)
F(1)-C(14)-C(15)	120.7(5)

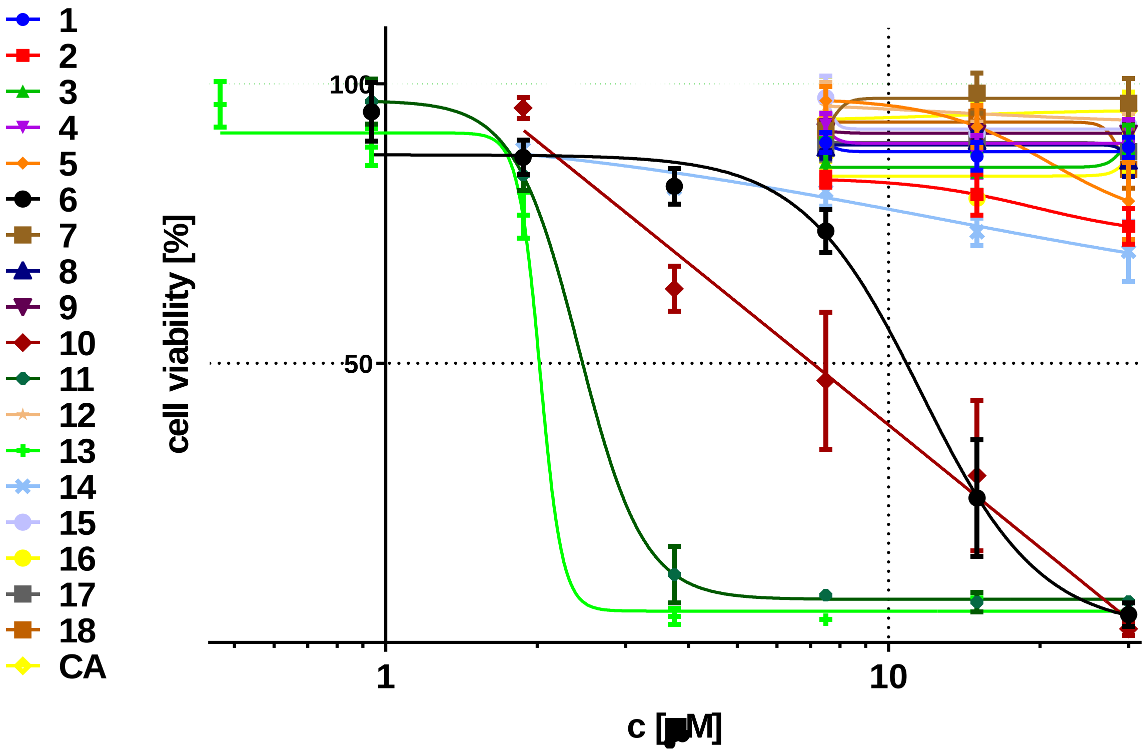

Figure S1. Cell viability determined by WST-1 assay. THP1-Blue™ NF-κB cells were incubated with given compounds, and the percentage of metabolic active cells was evaluated after 24 h. Each point represents the mean ± SEM of 3 independent measurements.

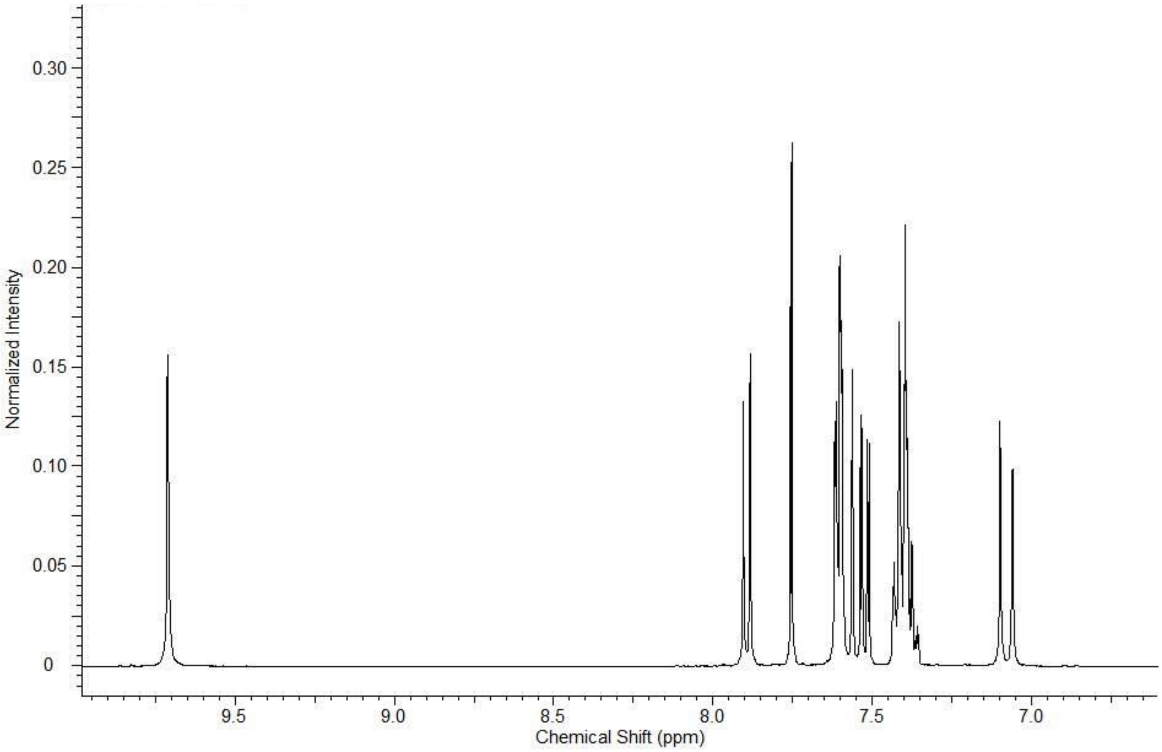

Figure S2. ^{1}H-NMR (DMSO-d_6) spectrum of (2*E*)-*N*-(4-bromo-2-chlorophenyl)-3-phenylprop-2-enamide (**16**).

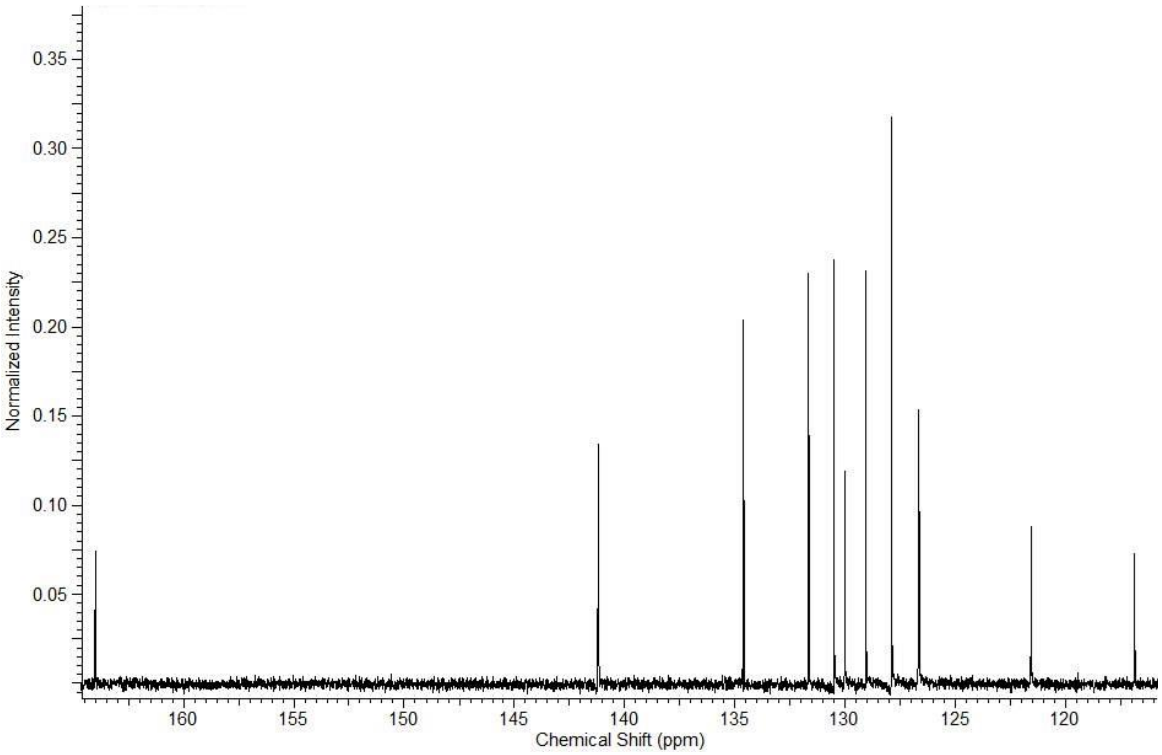

Figure S3. ^{13}C-NMR (DMSO-d_6) spectrum of (2*E*)-*N*-(4-bromo-2-chlorophenyl)-3-phenylprop-2-enamide (**16**).

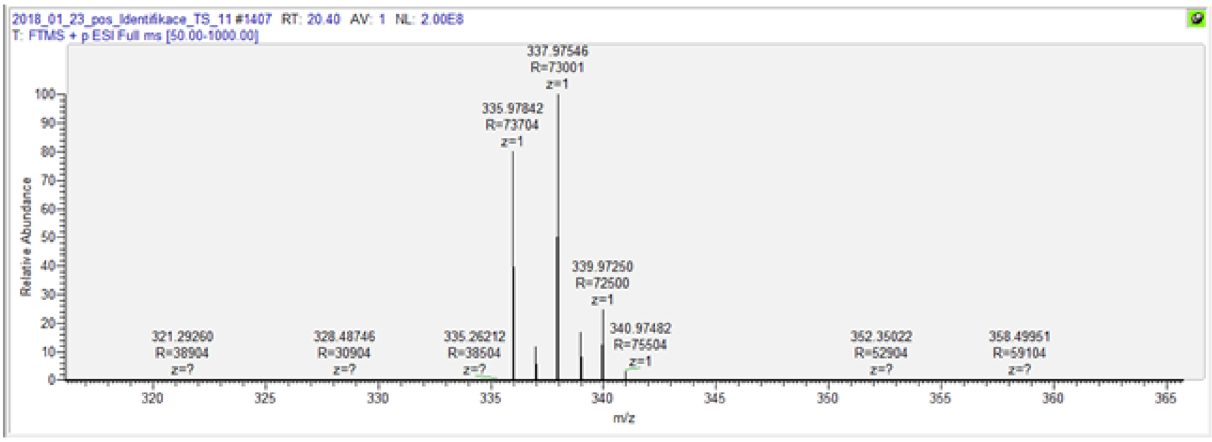

Figure S4. HR-MS record of (2*E*)-*N*-(4-bromo-2-chlorophenyl)-3-phenylprop-2-enamide (**16**).

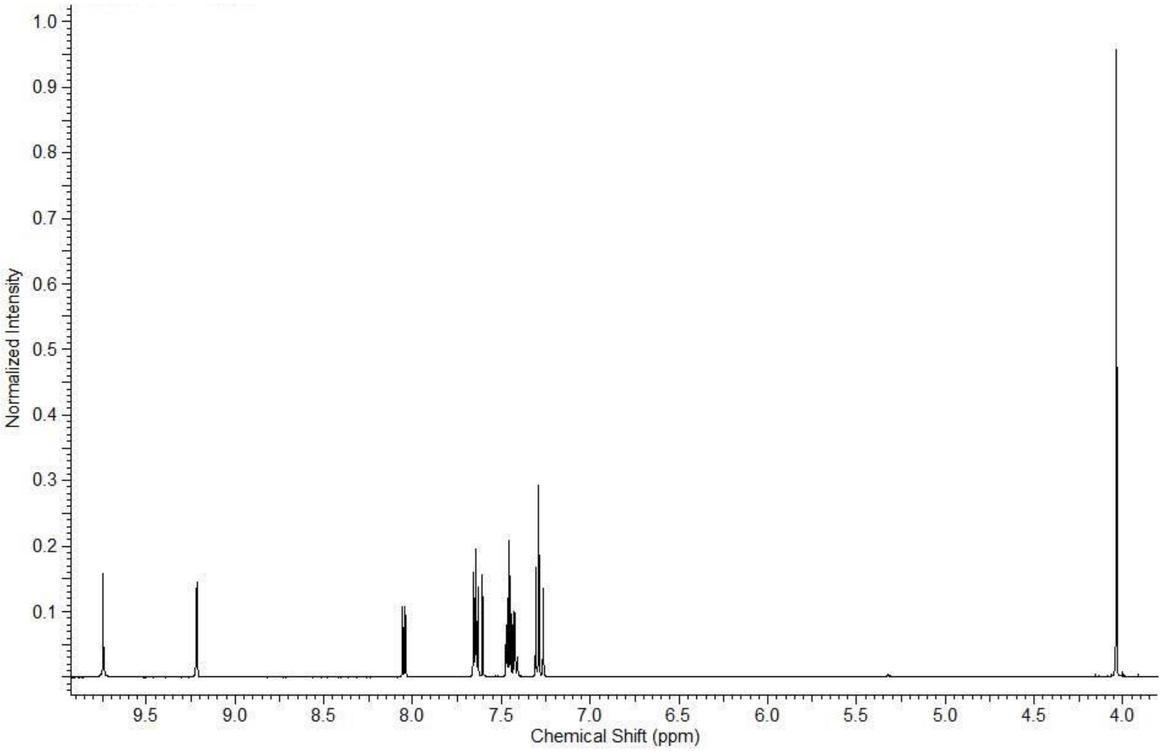

Figure S5. ^{1}H-NMR (DMSO-d_6) spectrum of (2*E*)-*N*-(2-methoxy-5-nitrophenyl)-3-phenylprop-2-enamide (**18**).

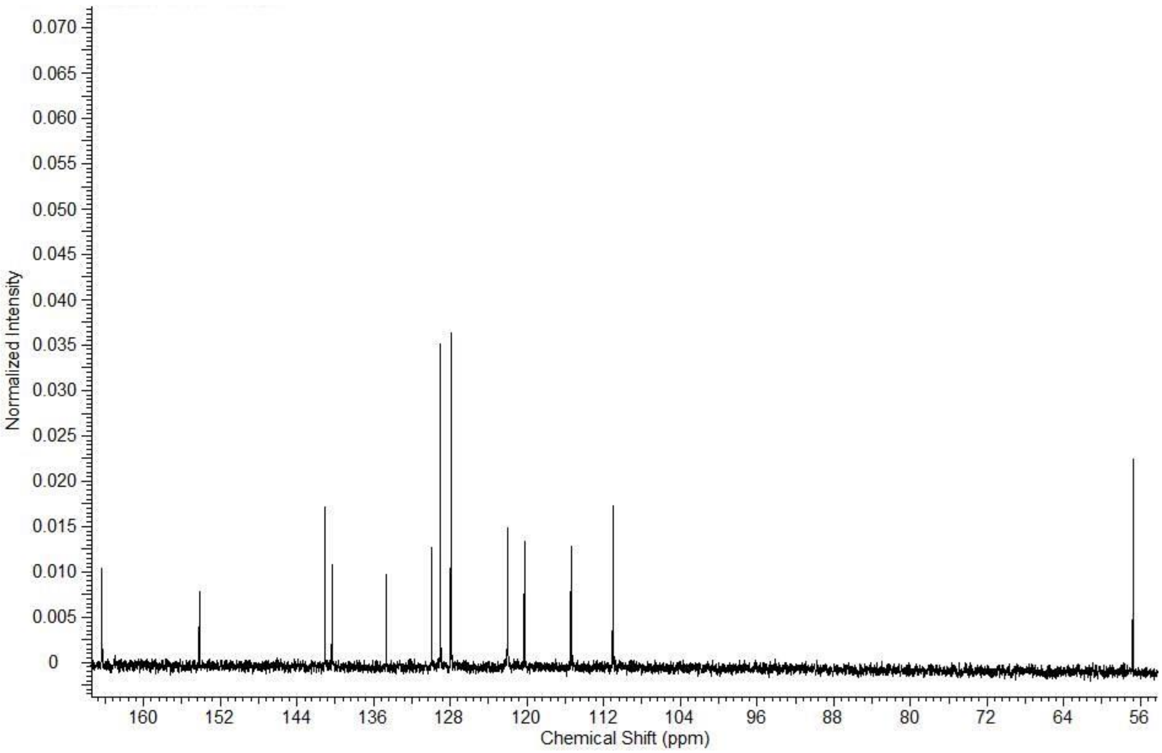

Figure S6. ^{13}C-NMR (DMSO-d_6) spectrum of (2*E*)-*N*-(2-methoxy-5-nitrophenyl)-3-phenylprop-2-enamide (**18**).

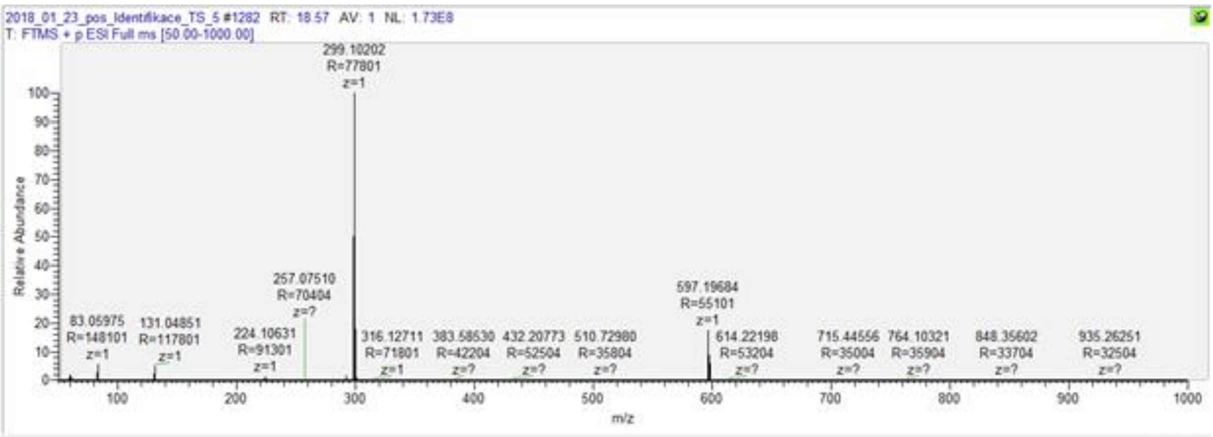

Figure S7. HR-MS record of (2*E*)-*N*-(2-methoxy-5-nitrophenyl)-3-phenylprop-2-enamide (**18**).
